# Assessing the health impacts of the urban expansion of small cities in China: A case study of Jiawang

**DOI:** 10.1371/journal.pone.0279470

**Published:** 2022-12-22

**Authors:** Yifu Ge, Zhongyu He

**Affiliations:** School of Architecture and Urban Planning, Nanjing University, Nanjing, China; Northeastern University (Shenyang China), CHINA

## Abstract

Empirical studies undertaken in developed countries have shown that urban expansion may exert both positive and negative impacts on residents’ health, depending on the planning strategy; however, the impact of rapid urban expansion on public health in developing countries is understudied. This paper takes Jiawang, China, as an example of rapid urban expansion and carries out a health impact assessment (HIA) on its regulatory detailed plan to better understand the interaction of the built environment and public health. We establish an HIA framework and select a series of indicators as health determinants. On this basis, we examine what impact the urban expansion will exert on the health equity of the residents by conducting a bivariate spatial autocorrelation. The finding shows that:1) Urban expansion produces positive health impact through the health determinants of public facilities, road transportation and land use. 2) Urban expansion will reduce health disparities between the old and new town and between the urban and suburban areas, especially between the old and new town. 3) The impact of expansion exerts on health equity will be generally positive. Low-income neighborhoods in the old town will significantly benefit from urban expansion in terms of road traffic and land use, but will not fully benefit in terms of public facilities. Low-income neighborhoods will no longer benefit from the accessibility to commercial facilities and will suffer from health inequities in terms of accessibility to healthcare facilities. 4) The government’s development strategy of emphasizing on an even distribution of public resources will unintentionally contribute to improving health equity. The significant promotion of health equity will mitigate the negative impacts of the previous urban development.

## 1. Introduction

Small city is the dominant urban administrative unit in China. According to the division standard of urban scale issued by the Chinese State Council in 2014, a city with a permanent population of less than 500,000 can be defined as a small city [1]. China’s city size distribution obeys the rank-size rule [2], with a larger number of small cities than medium-sized and large cities. China’s urbanization has advanced rapidly since the initiation of economic reforms in 1978, which triggered the largest population flow in history [3]. This urbanization has occurred at a faster pace than economic growth over the same period.

A unique characteristic of urbanization in China is that the urbanization of land is faster than that of the population [4]. Researchers believe that investing in low-cost land resources as the engine of local development is part of a long-term strategy to facilitate China’s economic development [5]. This conclusion seems especially likely given national policies that encourage the development of small cities alongside the development of agriculture and industry [6,7], which has led to the rapid expansion of small cities in the past 20 years and has played an important role in China’s urbanization process [8–10]. Meanwhile, rapid urbanization has also stimulated a series of socio-economic problems across China [11], such as traffic congestion, insufficient public services [12–15], excessive use of resources [16], climate change related issues [17–20], and environmental pollution [21–24]. In China’s small cities, there exists a strong contrast in spatial form between the new town and the old town, and between urban areas and suburban areas, which causes significant social differentiation [25,26]. Informal development, such as urban village and shantytown, often occurs without sufficient concurrent infrastructural development, resulting in a widespread lack of public services in some areas. The local industry tends to develop in pace with small city expansion, which contributes, to the problem of environmental pollution. Due to the manifold issues listed above, the physical activity level of residents in smaller cities is commonly lower than that of residents in larger cities, leading to less healthy lifestyles [27,28], and less awareness of individual healthfulness [29]. However, despite studies recording these issues, the health impact of small city expansion remains understudied and is often ignored by planners and local government.

Health impact assessment (HIA) is an analytical tool and research method that focuses on human health to work toward environmental justice and health equity [30]. According to the definition given by the World Health Organization in 1999, HIA is "a combination of procedures, methods and tools by which a policy, program or project may be judged in terms of its potential effects on the health of a population and the distribution of those effects within the population" [31]. HIAs are carried out across the globe and span many fields: the HIA of urban planning is an important branch of this research. HIA of urban planning has been conducted to assess the health impact of the comprehensive plan, as well as the plan in specific fields such as transportation plan [32], greenway plan [33], corridor plan [34], etc. HIA also explores the practices of urban design at different scales, and is deeply involved in the process of urban redevelopment.

Research on the impact of urban expansion on public health was first provided by an HIA conducted in Humboldt County, California, in the United States. According to the HIA, unrestricted growth and expanded development could cause decreased physical activity and social cohesion, and greater dependence on cars [30]. Currently, the deployment of HIA has mainly been carried out in developed countries. However, due to divergent urban morphology, urban planning regulations and socio-economic development conditions, the impact of urban expansion on health outcomes in developing countries such as China may be different and require further exploration.

In this paper, we use a regulatory detailed plan to establish a scenario for future urban expansion in China and study the connections between the regulatory detailed plan, the built environment, and public health. The aim of the current study is threefold: firstly, we aim to establish an HIA framework applicable to the evaluation of China’s small cities, and to examine the extent to which urban expansion will impact public health through a case study; secondly, we determine how the urban expansion will affect existing health disparities in the city by comparing the spatial differentiation of health determinants in the built environment before and after urban expansion; thirdly, we examine what impact the urban expansion will exert on the health equity of the residents by examining the correlation between housing prices alongside the distribution of health determinants.

## 2. Connections between urban planning, the built environment, and public health

The theoretical framework for this study is based on the theory of "urban health niche" [35], which is derived from the concept of an ecological niche and defines the urban health niche of an individual as a spatio-temporal manifestation of the causal agents and processes functioning at the micro, meso and macro scales. In each scale, the health niche can act as a subsystem, and the impacts of constituent factors are often synergistic and associated within and between subsystems.

**Fig 1:**
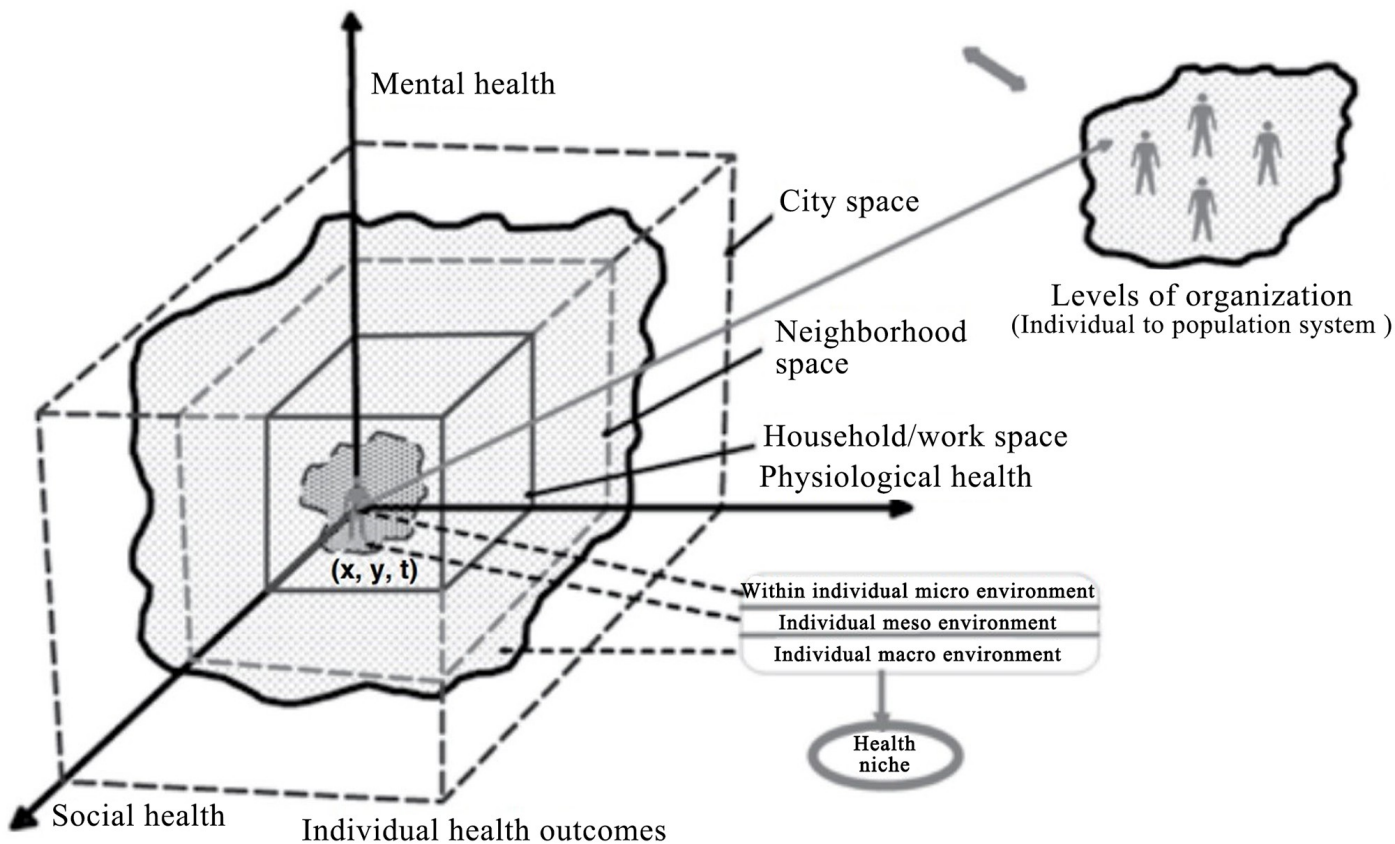
The conceptual framework of the urban health niche [35].

According to the theoretical model of the urban health niche, the impact of urban planning will be decipherable through multiple health-defining processes (Fig 1). As a policy that has a profound impact on public health, urban planning will first influence the urban health niche at the macro scale, which directly affects the built and natural environment at the neighborhood-level and city-level. Second, via the hierarchical transmission mechanism of the urban health niche, the impact of neighborhood-level change begins to impact the risk factors at the behavioral and lifestyle-level, which is the domain of risk factor epidemiology at the meso scale. Third, the micro scale encompasses individual-level processes, including physiologic and genetic epidemiology. When we order the impact of urban planning in this way, the interactions within and between the subsystem seem simple and linear. However, the interactions cannot, and are not, so simple and clear; indeed, the influence pathway that these elements occur along could be highly differentiated and complex. If we can define the influence path between different scales of the health niche, the evaluation of the health outcomes produced by urban planning can be realized.

There is wide ranging research establishing how the built environment affects individual behavior and living habits at the meso scale. Based on existing literature, we summarize the impact paths of urban planning on public health as obtaining public services [36], reducing negative health effects [37–41], promoting physical activity [42] and promoting social communication [43]. We define obtaining public services as improving convenience for urban residents seeking healthcare, recreational facilities, etc. Reducing negative effects means seeking to improve the quality of the built and natural environment, reduce the health risk exposure and the probability of accidental injury. Promoting physical activity means improving the intention, time and frequency of physical activity of residents. Promoting social communication means providing public places that induce social activities. Through these four pathways, researchers can track the role of urban planning in the control of space and how it translates into the health niche at the meso scale, which indirectly affects the individual health of the micro health position, and finally is reflected in physical and mental health.

## 3. Study area

Jiawang epitomizes the vast small city that dominates China’s geography. It is located in the northern Yangtze River Delta, a relatively underdeveloped area in one of the most developed regions in China. Jiawang has experienced rapid development in the past twenty years. Although its population only increased from 496,900 to 520,700 between 2000 and 2015, the ratio of agricultural households to non-agricultural households shifted from 65.28:34.72 to 44.50:55.50, indicating that at least 20% of the population underwent urbanization within those 15 years. During the same period, Jiawang’s GDP increased from 5.523 to 28.425 billion yuan, and recorded annual investments in fixed assets increased from 818 million yuan to 21.965 billion yuan.

**Fig 2:**
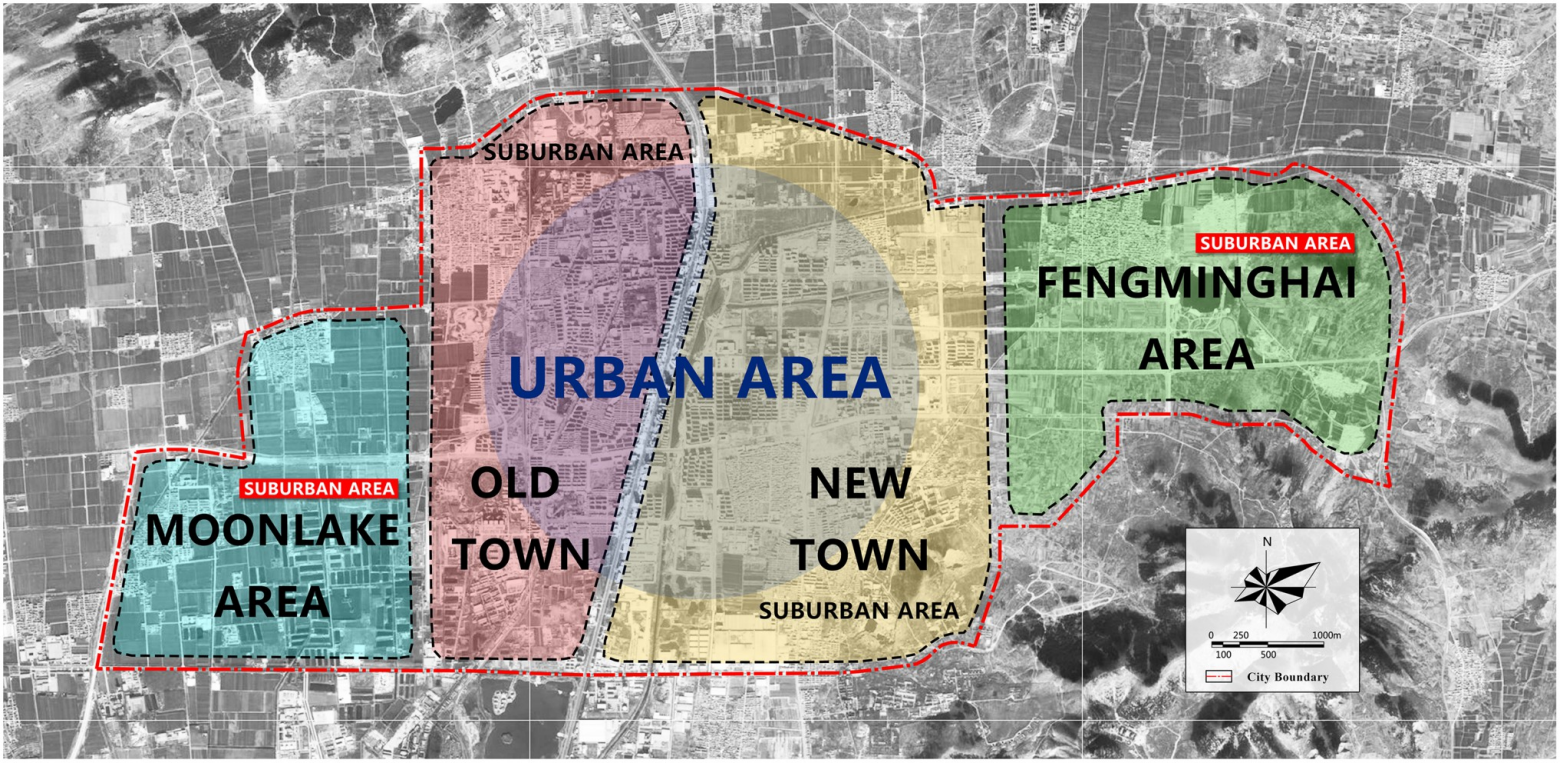
The spatial structure of Jiawang in the regulatory detailed plan.

By the end of 2016, the population within Jiawang’s urban area totaled 310,000. The development of the central urban area is not completed. A large number of undeveloped areas still exist, and several villages have not been developed. We create a figure to present the spatial structure of Jiawang (Fig 2), with data collected from the June 06, 2018 version of USGS World Imagery map (https://usgs.maps.arcgis.com/home/item.html?id=474c65ab3e1941468511785495eb8987). Due to Jiawang’s rapid urbanization, a new town emerged on the east side of the old town (Fig 2), which is divided from the old town by a north-south expressway. The old town has experienced a long period of development, resulting in the concentration of public service facilities. However, the old town has some structural issues. The road transportation network is incomplete, the land layout is chaotic, and a large number of industrial areas are scattered throughout the old town, making it less easily navigable. Administrative centers and large-scale educational facilities were moved to Jiawang’s new town, however, there is still a lack of public service facilities in the new town.

The regulatory detailed plan concerned in this study is a unique type of urban planning in China. It was created based on references to land zoning control methods (mainly zoning) combined with China’s urban management system. A regulatory detailed plan follows a master plan with a focus on land use control. This type of urban planning specifies land use types, land use intensity and environmental design as the basis for urban planning management. In addition, by providing regulations for land lease and transfer, a regulatory detailed plan directly guides urban development and construction. This form of systematic planning has a comprehensive impact on the built environment by specifying an ordinance that all development projects should follow; therefore, a regulatory detailed plan can be used as a projection of future urban expansion. In Jiawang, the regulatory detailed plan divides the city into multiple control units, and determines land use, building codes, transportation and public facility availability for each control unit. The control unit is deployed as the unit for spatial analysis in this paper (see S1 Fig for control units’ number and distribution).

In September 2018, Jiawang initiated the regulatory detailed plan, and the draft was submitted to the Planning Committee in January 2019. In this version of the plan, the urban boundary was expanded to encompass both eastern and western suburban areas. Post expansion, Jiawang will be comprised of four parts: the old town, the new town, the Moonlake area and the Fengminghai area. The urban area is located in the central part of the old town and the new town (Fig 2). The suburban area includes the peripheral areas of the old town and new town, as well as the Moonlake and Fengminghai area. In the regulatory detailed plan, the old town is supposed to act as a comprehensive development area, whereas the new town will provide living space for residents, the Moonlake area will focus on creative industries, and the Fengminghai area will house leisure and health services. The total population of the urban area is expected to reach 400,000 by 2035.

## 4. The selection and measurement of the health determinants

As explained above, the regulatory detailed plan is regarded as a way of representing future urban expansion. Three dimensions, namely public facilities, road transportation and land use, are the major concerns of the plan that may have a health impact at the meso level. Public facilities put forward quantitative configuration requirements for various public facilities, including size and spatial layout. Road transportation puts forward control regulations for traffic activities, including the design of urban traffic network and the traffic organization of land parcels. Land use includes the regulations of land development and environmental capacity. Road traffic and land use interact with each other, which is reflected in that the road traffic network affects the land use pattern, while land use affects the traffic structure to a certain extent. As a specific land use function, the use of public facility land is planned based on the surrounding land use and road traffic affects its accessibility.

Within each dimension, a series of built environment indicators (health determinants) at the neighborhood-level and city-level are selected to assess whether and to what extent the plan will improve public health, compared with the current status (Table 1). The selection of the indicators is based on a broad literature review that identified built environment factors that were frequently reported to be associated with public health and related health evidence (see S1 Appendix).

**Table 1:** Indicators of the HIA of Jiawang’s regulatory detailed plan.

Dimensions	Health determinants	Influence path	Impact	Measurement
**Public facilities**	Proportion of green space	①②③④	+	the proportion of green space in the control unit
	Per capita green space	②③④	+	the per capita green area in the control unit
	Service coverage of educational facilities (Kindergartens, primary schools and secondary schools)	②③	+	the proportion of residential areas within the service range of educational facilities
	Accessibility to green space	②③④	-	the average travel time
	Accessibility to recreational facilities (Cultural facilities, sports facilities, squares and other types of facilities)	①③④	-	the average travel time
	Accessibility to healthcare facilities (Medical and health facilities, public welfare facilities, rehabilitation facilities)	①	-	the average travel time
	Accessibility to commercial facilities	①③④	-	the average travel time
**Road transportation**	Road network density	②③	+	the total length of roads in the control unit divided by the total land area
	Intersection density	②③	+	the number of road crossings in the control unit divided by the total land area
**Land use**	Land use mix	②③	+	the entropy value of the land use types in the control unit
	Residential density	③	+	the proportion of residential area and occupied land area in the control unit
	Residential environment	③④	-	the residential area with poor construction quality and affected by excessive noise in all residential areas in the control unit

①obtaining public services; ②reducing negative health effects; ③promoting physical activity; ④promoting social communication.

## 5. Methods and data

For school coverage, according to China’s regulations on the allocation of educational facilities, the service scope of kindergartens, primary schools and secondary schools is determined as 500m, 1000m and 1500m respectively. The service area is simulated based on the road network model built with ArcGIS 10.5 software. The data of various educational facilities is obtained from the area and distribution in the current and planned land use map.

For accessibility, based on the road network model built in ArcGIS 10.5 software, the OD matrix is used to calculate the shortest travel distance and travel time. The travel probability from each junction to each destination is calculated, and the average weighted travel time is calculated to obtain accessibility. The road network is acquired from the planned land use map. The population in the plan is estimated based on the current residential density and the residential land area in the plan.

In our analysis of the residential environment, the impact of construction quality and noise pollution is considered. We do not consider air quality because it is lack of variation due to the relatively small size of the city. First, we calculate the total residential area in each control unit that is designated a poor construction quality according to the land classification in the current land use map. Second, we calculate the total area in each control unit suffering from noise pollution. The noise sources are designated to be industrial land, logistics land and public transport station, and the noise level is set to be 79dB by averaging the noise levels of different types of vehicles. Based on the standard for urban residential area planning, the noise level in a residential area shall not exceed 50dB. According to the natural attenuation of noise, a 200m buffer zone is required for a 29dB (79dB-50dB) noise reduction. Therefore, we calculate the total area of residential land that is within the 200m buffer of the above noise sources in each control unit. The data of the residential area, industrial land, logistics land and public transport station is acquired from the current and planned land use map.

We use land use entropy to evaluate land use mix. The land use mixed degree *M*_*i*_ represents the level of mixed distribution of different land use types within a certain range [44]. The calculation formula is as follows:

$$M_{i}=\frac{-\sum_{K=1}^{K}P_{k,i}ln(P_{k,i})}{ln(-K,i)}$$ (1)

*K* is the number of land use types. *P*_*k*,*i*_ is the area proportion of K land in control unit *i*. The land use mix values usually range from 0 to 1, with 0 representing a homogeneous, single land use environment, and with 1 representing a perfectly heterogeneous neighborhood comprising all possible permutations and combinations of land use categories. The data of land use type is acquired from the current and planned land use map.

We use a bivariate treatment of spatial autocorrelation to evaluate health equity. The notion of geographies of need by Harvey (1973) suggests that localities with a larger presence of disadvantaged residents are in need for better access to public services and goods [45]. Therefore, in our study, we define “health equity” as health resources should be allocated in favor of disadvantaged social groups. Income is the most widely adopted indicator for measuring socioeconomic status. However, obtaining data of residents’ income is difficult. Due to the significant correlation between housing prices and income [46], we use housing prices instead. A bivariate spatial autocorrelation is carried out to analyze the spatial association between housing prices and various health determinants. The housing price data of Jiawang is collected on the "Anjuke" website(https://xuzhou.anjuke.com/?from=) in 2021. We collect 249 residential areas. Among them, 145 residential areas are matched to the map using GIS. We retain a total of 97 residential areas out of a possible 145 by filtering out repeated addresses, residential areas that cannot be accurately located and residential areas located outside the study area. The distribution of Jiawang’s housing prices is obtained through kriging interpolation, and the average housing prices of the control units are calculated (see S1 File for average housing prices of each control unit).

Using GeoDa software, we perform bivariate spatial autocorrelation analysis. The queen contiguity approach with the first order of neighbor was chosen to manage the spatial weights. Moran’s Index and cluster maps are obtained through bivariate spatial autocorrelation measures. Moran’s Index ranges from −1 to 1, with 0 indicating a completely random spatial arrangement. Four types of spatial association can be identified in the cluster maps, which are High-High, Low-Low, High-Low and Low-High. High-High and Low-Low types indicate a positive spatial association between one unit and its neighbor units, while High-Low and Low-High indicate a negative spatial association between one unit and its neighbor units.

## 6. Results

### 6.1 Overall assessment of the change of health determinants

We calculate the average value of all control units for the current and projected expansion scenarios, and calculate the percentage change between these two scenarios (Table 2).

**Table 2:** The average values of the indicators of 71 control units.

Dimensions	Health determinants	Current	After expansion	Percentage change
**Public facilities**	Proportion of green space (%)	7.4193	16.1125	1.1717
	Per capita green space (m2)	15.8725	22.1873	0.3979
	Service area of educational facilities (kindergartens) (%)	4.7239	15.9897	2.3849
	Service area of educational facilities (primary schools) (%)	7.9721	19.8069	1.4845
	Service area of educational facilities (secondary schools) (%)	23.0160	5.1891	1.2546
	Accessibility to green space (h)	0.0401	0.0050	-0.8765
	Accessibility to recreational (h) facilities	2.0370	0.4697	-0.7694
	Accessibility to healthcare facilities (h)	0.4808	0.5956	0.2386
	Accessibility to commercial facilities (h)	0.0331	0.0119	-0.6418
**Road transportation**	Urban road network density (km/km2)	2.9026	8.1621	1.8120
	Intersection density (/km2)	10.2664	21.9459	1.1373
**Land use**	Land use mix (/)	1.3400	2.1794	0.6264
	Residential density (%)	25.0755	41.2980	0.6469
	Residential environment (/)	122.1972	7.4648	-15.3698

As shown in Table 2, the regulatory detailed plan will significantly improve the health outcomes of the built environment. Among the 14 indicators, only the accessibility to healthcare facilities is negatively impacted. However, there is a great disparity between the percentage change of different health determinants. Among them, the most significant shift is the residential environment. Compared with the current situation, the residential environment value will decrease by 15.3698 times. In addition, the service area of all three types of educational facilities will improve significantly as well.

### 6.2 Spatial differentiation of health determinants

**Fig 3:**
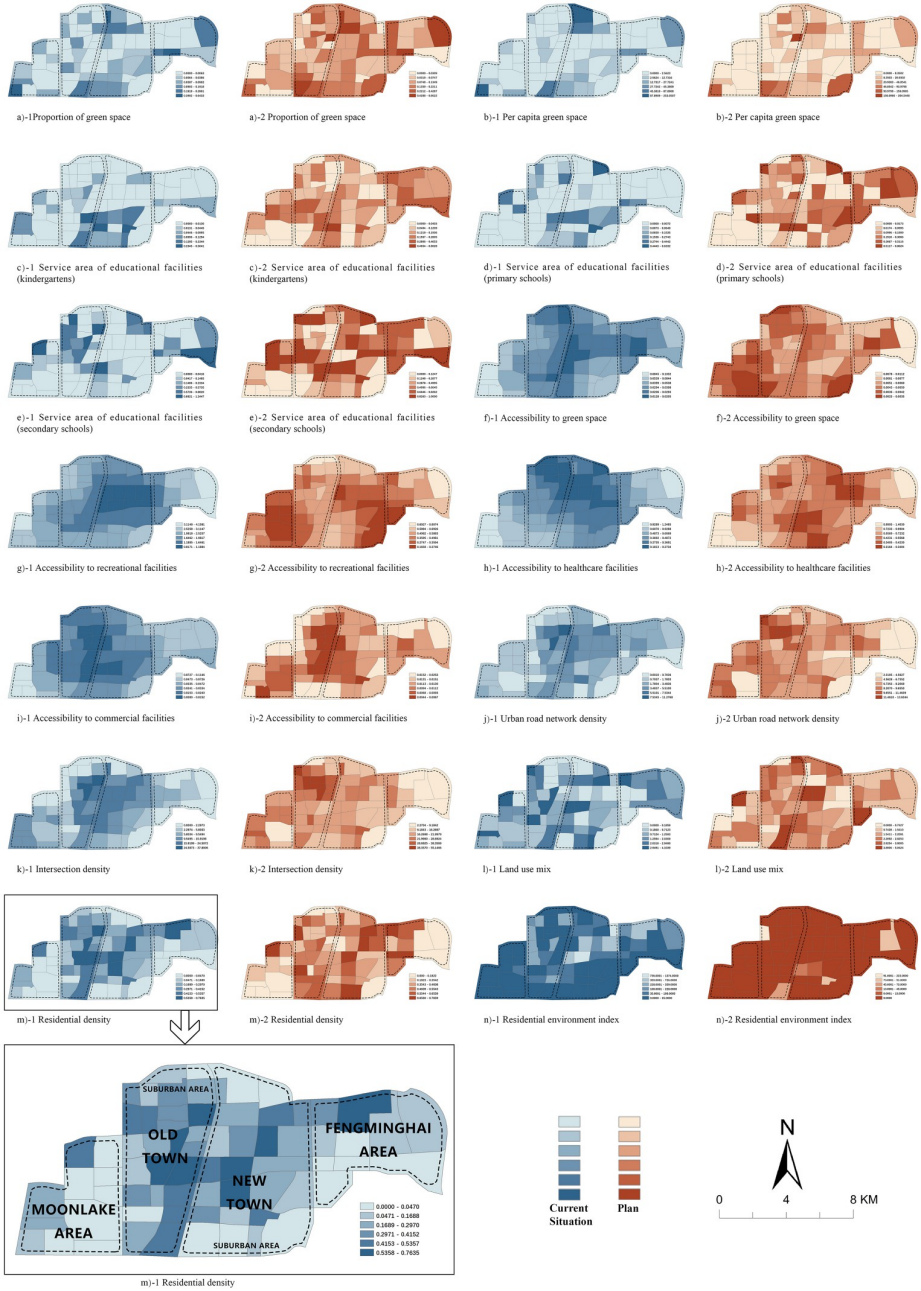
Value of the health determinants across all control units.

The values of the 14 health determinants for the 71 control units before and after urban expansion are shown in Fig 3. We focus on the spatial differentiation between control units in the current situation and after expansion.

Spatial differentiation is pronounced both in the current situation in Jiawang and in the improvements seen with the regulatory detailed plan. These spatial differentiations are thus also reflected differently in the health determinants. The spatial differentiation is mainly reflected between the urban and the suburban areas. In addition, it is also reflected between the old town and the new town in some indicators. For instance, in the current situation, significant spatial differentiation exists across the public facilities, road transportation and land use. Accessibility to green space, recreational facilities, healthcare facilities and commercial facilities shows significant mono-center agglomeration in the new town. Accessibility to educational facilities is also highly concentrated in specific areas, such as the urban area of the old town and just south of the new town. Green spaces are relatively concentrated in the suburban area. The urban road network density and intersection density is higher than that in the underdeveloped suburban area. Significant spatial differentiation exists in land use between the urban area and suburban area as well.

In the plan, the spatial differentiation of health determinants between urban areas and suburban areas will continue to exist, only weakened compared with the current situation. Green space related indicators still will be higher in the suburban area. Accessibility to green space, educational facilities, recreational facilities and healthcare facilities will all show patterns of spatial differentiation in Jiawang, but will not highly concentrated in some areas. Notably, accessibility to commercial facilities will be highly agglomerated in some areas such as urban areas, the Moonlake area, and the Fengminghai area. The spatial layout of land use and road traffic will be similar, and will be relatively higher in the urban area.

### 6.3 Correlation between housing price and health determinant

Based on the HIA of the regulatory detailed plan, we carry out bivariate spatial autocorrelation to explore the possible spatial association between housing prices and health determinants, as well as to examine the differences between the current situation and the post-expansion plan. Moran’s Index between housing prices and the health determinants is shown in Table 3 (see S1 File for data of housing prices and health determinants).

**Table 3:** Moran’s Index between housing prices and the health determinants.

		Current				After expansion		
Dimensions	Health determinants	Moran’s I	P-value	Z-value	Moran’s I		P-value	Z-value
**Public facilities**	Proportion of green space	0.1661***	0.0100	2.9321	-0.1885***		0.0100	-3.3228
	Per capita green space	0.1558***	0.0100	3.3638	-0.1101***		0.0100	-2.3306
	Service area of educational facilities (kindergartens)	-0.0296	0.3300	-0.4300	-0.1351**		0.0200	-2.3301
	Service area of educational facilities (primary schools)	-0.0408	0.2900	-0.6849	-0.1459**		0.0200	-2.7035
	Service area of educational facilities (secondary schools)	0.0288	0.2500	0.5929	-0.2046***		0.0100	-3.7685
	Accessibility to green space	-0.1487***	0.0100	2.5907	0.3792***		0.0100	-8.6726
	Accessibility to recreational facilities	0.0592	0.1600	-0.9442	0.1544***		0.0100	-2.6158
	Accessibility to healthcare facilities	0.0869*	0.0800	-1.7317	-0.0836***		0.0100	2.0080
	Accessibility to commercial facilities	0.1023*	0.0900	-1.6738	0.0656		0.1200	-1.2404
**Road transportation**	Urban road network density	0.0324	0.3000	0.5903	-0.3841***		0.0100	-6.2853
	Intersection density	-0.1004**	0.0400	-1.7498	-0.3827***		0.0100	-7.0147
**Land use**	Land use mix	0.0877*	0.0700	1.4914	-0.0758*		0.0700	-1.4302
	Residential environment	-0.0683	0.1300	-1.0009	-0.0791**		0.0500	-1.5323
	Residential density	-0.0693	0.1100	-1.4854	-0.0381		0.3100	-0.4917

*** significant at 0.01 level.** significant at 0.05 level.* significant at 0.1 level.

In the current situation, half of the 14 bivariate Moran’s Indexes are statistically significant, and the number will increase to 12 after expansion. Bivariate Moran’s Index of housing prices and commercial facility accessibility is only significant in the current situation. Spatial correlation of housing prices and six health determinants will be only significant in the plan, which are service area of educational facilities (all three determinants), accessibility to recreational facilities, urban road network density, and residential environment. Among them, recreational facility accessibility will be positive while the other indicators will all be negative.

The results of bivariate Moran’s index indicate that the change of spatial association between housing prices and significant health determinants will occur in three ways after expansion: 1) changing from a positive association currently to a negative one in the plan, including land use mix, accessibility to healthcare facilities, proportion of green spaces and per capita green space. This is the most common pattern of change. 2) Changing from a negative association currently to a positive one in the plan, which only occurs to housing prices and green space accessibility. 3) The bivariate Moran’s Index of housing prices and intersection density is negative with larger absolute values in the plan, thus indicating that they will have a stronger negative association.

**Fig 4:**
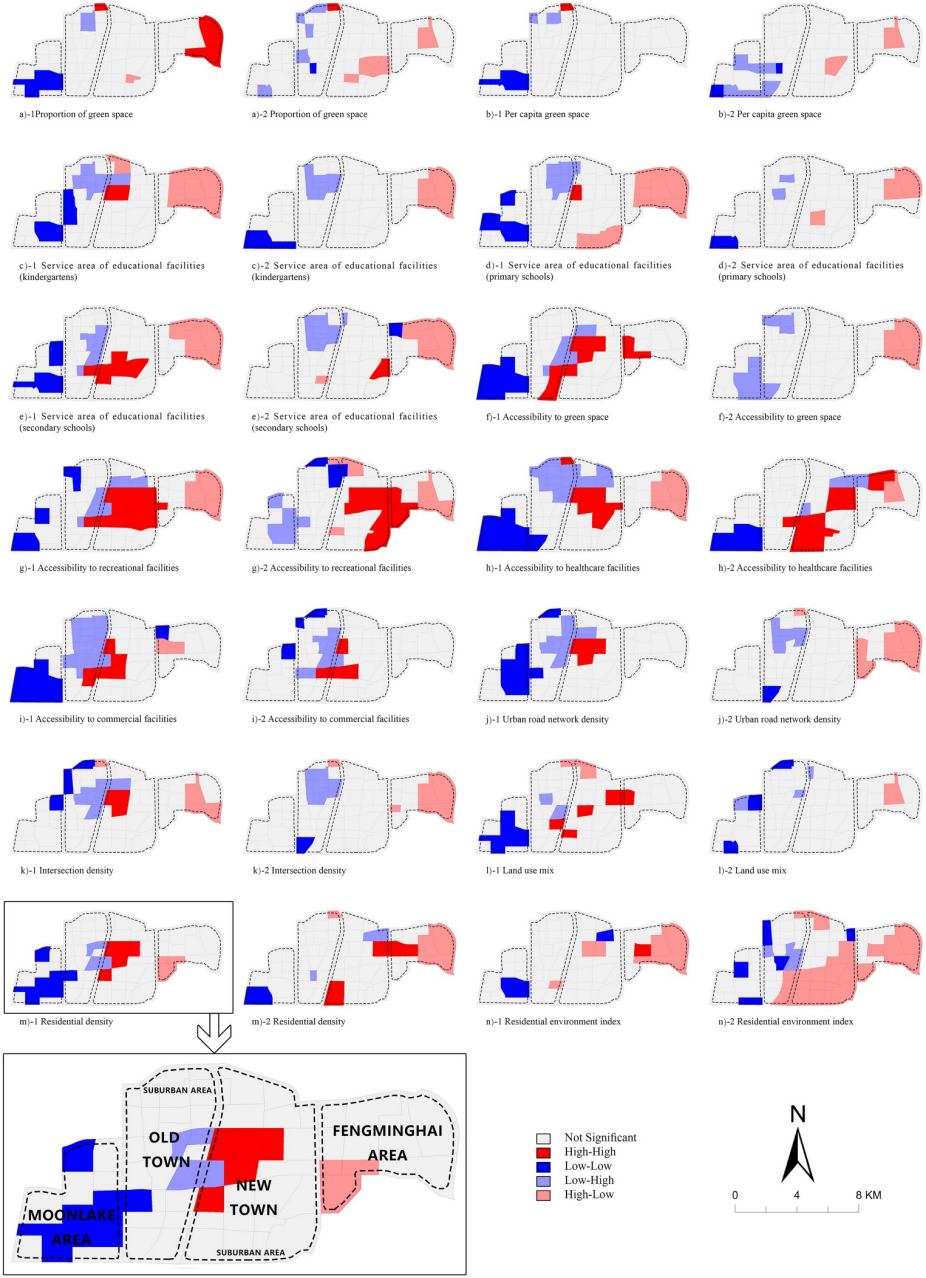
Cluster maps showing local spatial autocorrelation analysis between housing prices and health determinants.

As is shown in Fig 4, spatial cluster association exists between housing prices and health determinants, both in the current situation and after expansion under the regulatory detailed plan.

In the current situation, Low-Low association is mostly found in the Moonlake area, while High-High association is mostly found in the urban area of the new town, indicating that in these regions, wealthier neighborhoods can enjoy a healthier built environment while poorer neighborhoods suffer from a lack of health resources. Low-High association mainly appears in the urban area of the old town and High-Low association mainly appears in the Fengminghai area in the suburban.

After expansion, fewer positive association and more negative association patterns including High-Low and Low-High are shown. Negative association will be found between housing prices and almost all health determinants. Most High-Low neighborhoods will be situated in the Fengminghai area of the new town while Low-High neighborhoods will be situated in the central area of the old town, indicating that poorer neighborhoods will benefit greatly from the expansion. Both High-Low and Low-High distributions will be relatively concentrated. In some cases, High-Low neighborhoods will also appear outside the Fengminghai area. For example, High-Low blocks will be situated in the south of the new town in terms of housing prices and proportion of green space, per capita green space and service area of educational facilities. Low-High neighborhoods will appear in the Moonlake area regarding three green space relating health determinants and accessibility to recreational facilities.

High-High association will be found between housing prices and accessibility to recreational facilities, healthcare facilities and commercial facilities, and these will mostly appear in the new town. Low-Low association will still mainly appear in the Moonlake area. However, compared with the current situation, Moonlake’s Low-Low association will only be found between housing prices and accessibility of healthcare facilities. Also, the number of Low-Low control units will be less than that of the current situation.

According to the cluster maps, health inequities between areas with different housing prices will continue to exist after the enaction of the plan, however, the spatial association patterns will change. The High-Low and Low-High associations in the plan will be strengthened, while the High-High and Low-Low associations will be weakened.

## 7. Discussion

### 7.1 Health impact of Jiawang’s urban expansion

According to the analysis of various determinants, the built environment regarding public health will be significantly improved after urban expansion. This improvement will be mainly reflected in terms of better access to public facilities, more appropriate land use and less congested road transportation.

In the process of urban expansion, the road network is often the primary focus for urban planners. Therefore, it is common to see a positive impact on public health in relation to road transportation. In the plan, the complicated and chaotic road network in the urban area of the old town is redesigned, and systematically extended to the suburban area. Therefore, in terms of road traffic, network density and intersection density will see significant improvement.

As the road network is adjusted, so too will land use be reassessed and reallocated simultaneously. The rearrangement of various types of land use in the plan will have a significant impact on land use patterns in Jiawang overall. Currently, land use is structured in a relatively simple way in the remote suburban areas of Jiawang: the plan will increase the land use mix in these areas. With the increasing demand for residential areas, residential land will expand. As a result, the residential density in the suburban area will increase. The residential land with poor environment in the old town will be redeveloped, the overall residential environment sees beneficially shifts.

The plan also pays attention to the distribution of public facilities. For example, with the growth of population and the expansion of residential areas, recreational facility and commercial facility accessibility will be greatly improved, especially in the suburban area. The plan for green space mainly focuses on a large-scale green space development in the suburban area since, with the development and utilization of the suburban area in the expansion, the amount of green space in the suburban area will decrease. The educational facilities are also rearranged by delimiting the service area. In doing so, the service area of various educational facilities will be significantly improved, and the distribution of educational resources will be more even across all areas.

### 7.2 Mitigation of health disparity

Urban expansion in the plan will change the spatial differentiation patterns across various health determinants. This spatial differentiation corresponds to the expansion process of Jiawang, between the old town and the new town, and between the urban area and the suburban area.

Currently, the spatial differentiation between the old town and the new town mainly exists in the realm of public facilities. The main reason is that during the urbanization process, the government vigorously supports the development of the new town through superior public resource allocation. The river and surrounding natural environment that originally separated the old town from the new town will provide better access to green space in the new town. The urban structure will revolve after the planned expansion, resulting in significant functional differences between the old town and the new town. New town and old town will no longer be separated from each other, but functionally interconnected. The spatial differentiation between the old town and the new town will thus be greatly weakened.

The spatial differentiation between the urban area and the suburban area lies in almost all health determinants of public facilities, road transportation and land use. Currently, this is mainly due to the mono-central structure of the urban area. The planned expansion will still result in relatively low residential density in the suburbs while the allocation of public facilities will be enhanced in this area. The spatial differentiation between the urban area and the suburban area will be reduced compared to the current situation, such as accessibility to various types of public facilities. The almost ubiquitous gap between the urban area and the suburban areas can be regarded as a reasonable decision based on the efficiency of resource allocation and a typical phenomenon in the expansion of small cities [47,48].

### 7.3 Health equity issue

Given the health disparities existing in the current and after expansion, our research focused on the possible health inequities and how particular subgroups might be affected. The current bivariate Moran’s Index suggests low-income neighborhoods suffer from health inequities in several health determinants, especially in terms of public facilities. In the current situation, high housing price concentrations are associated with land use mix, green space accessibility, proportion of green space and per capita green space, whereas low housing price concentrations are associated with healthcare facilities accessibility, commercial facilities accessibility and intersection density.

Compared to the current situation, expansion influences health equity among several health determinants. Low-income neighborhoods will benefit from the improvement of the amount of green space, green space accessibility, service area of educational facilities, recreational facility accessibility, urban road network density, intersection density, residential environment and land use mix. Among them, the improvement of green space will be relatively more significant. However, it is unlikely that urban expansion will have a positive impact on health equity in all respects. Low-income neighborhoods will no longer benefit from accessibility to commercial facilities after expansion. Low-income neighborhoods in the old town will suffer from inequities in accessibility to healthcare facilities.

Overall, the expansion will result in significant improvements in health equity. Results suggest that low-income neighborhoods will not be disadvantaged in land use and road transportation, but will not fully benefit from public facilities. We speculate that the development strategy will be the possible explanation for this positive health outcome. The development strategy of emphasizing on an even spatial distribution of public resources is deeply involved in the regulatory detailed plan, as well as the functional positioning of different areas. The functional positioning of old town to provide public services will give low-income neighborhoods an advantage in various health resources such as recreational facilities and educational facilities. Besides, the compact urban form of the old town will unintentionally benefit the low-income groups in terms of road traffic and land use. Therefore, the improvement of health equity is not an entirely planned outcome, low-income groups will be benefiting from the urban expansion in an unintentional way. The current housing price differentiation was exacerbated by the government’s development strategy in previous expansions, including the prioritization of improving public facilities, the natural environment and the living space in the new town. Rather than saying health equity will be greatly improved by expansion, we argue the fact is that the health inequity left by the previous rapid urban development will be partially remedied.

The association between housing prices and health determinants is an issue worth discussing. Housing is a commodity with high demand, and housing prices are closely related to resident income level and quality of life [49]. Health equity is usually measured through the relevant differences between better- and worse-off social groups [50]. Various associations between income level and health determinants are reported in different countries. Researches in China have shown that low-housing price areas may have certain advantages in density, street connectivity and land use mix [51,52]; on the other hand, a less healthy food environment is usually correlated with these areas, and it is more difficult to obtain access to public and private recreational facilities [53–55].

Contrastingly, in Toronto, Canada, it is observed that housing prices are negatively correlated with accessibility and positively correlated with distance to transit stops, cultural centers and schools [49]. For big cities in China that have experienced several rounds of urban expansion, most health determinants have a positive relationship with housing prices [56–58]. However, some exceptions are found in a few cities. In Xiamen, the closer to the hospital you move, the lower the housing prices are [57]. In Beijing and Wuhan, housing prices are found to be positively associated with the distance between supermarkets and/or vegetable farms and residential areas [56,58], which may be due to the negative externalities of these facilities. For a small city like Jiawang, the health impact of urban expansion will be even more profound, as the expanding area is fairly large compared with the current urbanized area.

### 7.4 Outlook

Although numerous studies have provided both theoretical and empirical evidence on urban planning’s impact on public health, no universal framework of HIA for urban planning has been established, largely due to the problem-driven nature of HIA in practice. We argue there is no one-size-fits-all HIA method and its implementation should be case specific. For instance, one limitation of our framework is that we did not consider the weight of the health determinants. Perhaps a more convincing weight distribution can be obtained through meta-analysis as propose [59], by assessing the weight average elasticity between travel and build environments, which can be used as a reference point when constructing the HIA index system. Nevertheless, the consideration of income inequality provides a new perspective for assessing the public health impact of urban planning. Considering the availability of data, we used the current housing prices in the analysis of the plan. However, housing prices will change dynamically with the expansion, which should be considered in future HIA research.

## 8. Conclusion

The regulatory detailed plan of Jiawang reflects the typical urban expansion process seen across China as small cities continue to rapidly expand. To our best knowledge, this is the first HIA to evaluate the health impacts of urban development in small cities in China. The results are as follows:1) According to the health impact assessment of the urban expansion that the regulatory detailed plan outlines, there will be positive health effects across public services, land use and road transportation when a plan is in place. 2) The spatial differentiation pattern of health determinants will change after expansion, as the health disparities between the old town and the new town will be greatly alleviated, and the gap between the urban and the suburban areas will narrow. 3) Health equity will be greatly promoted after expansion. Low-income neighborhoods usually will not be disadvantaged in road transportation and land use, but may suffer from health inequities in healthcare facility access with regard to public facilities. 4) It is the previous rapid urban expansion that has exacerbated the housing price differentiation. The improvement of health equity is an unintentional health outcome of the government’s development strategy. The positive impact on health equity that low-income neighborhoods will enjoy is a by-product of urban expansion.

For small towns in China, urban expansion will have significant positive health impacts on land use and road transportation, but may have negative impacts in terms of public facilities. Therefore, we put forward the following suggestions for small cities’ expansion in China:

First of all, more attention should be paid to the dynamic impact of expansion on residents’ spatial distribution before making decisions, and a more reasonable spatial association should be constructed at a larger scale in the process of expansion. Secondly, the differences of functional positioning between areas often lead to spatial differentiation in public facilities, which may exert negative health impacts on low-income neighborhoods due to allocation of public facilities. A series of measures can be taken, such as improving the allocation of recreational and healthcare facilities, so as to provide residents with diversified health-promoting public facilities. Thirdly, few health impact assessments have been carried out in urban planning in developing counties such as China. It is recommended that health impact assessment be applied to analyze the current health disparities and health inequities and to address health issues resulting from the rapid urbanization.

## Supporting information

S1 Fig Map of control units’ number and distribution. (TIF)

S1 File Housing prices and health determinants of the control units. (CSV)

S1 Appendix Literature review for the framework of the health impact assessment. (DOCX)
