# Supplementary figures and images for: Assessing the health impacts of the urban expansion of small cities in China: A case study of Jiawang
Source: PLoS One. 2022 Dec 22;17(12):e0279470. doi: 10.1371/journal.pone.0279470 (PMC9778500; doi:10.1371/journal.pone.0279470)

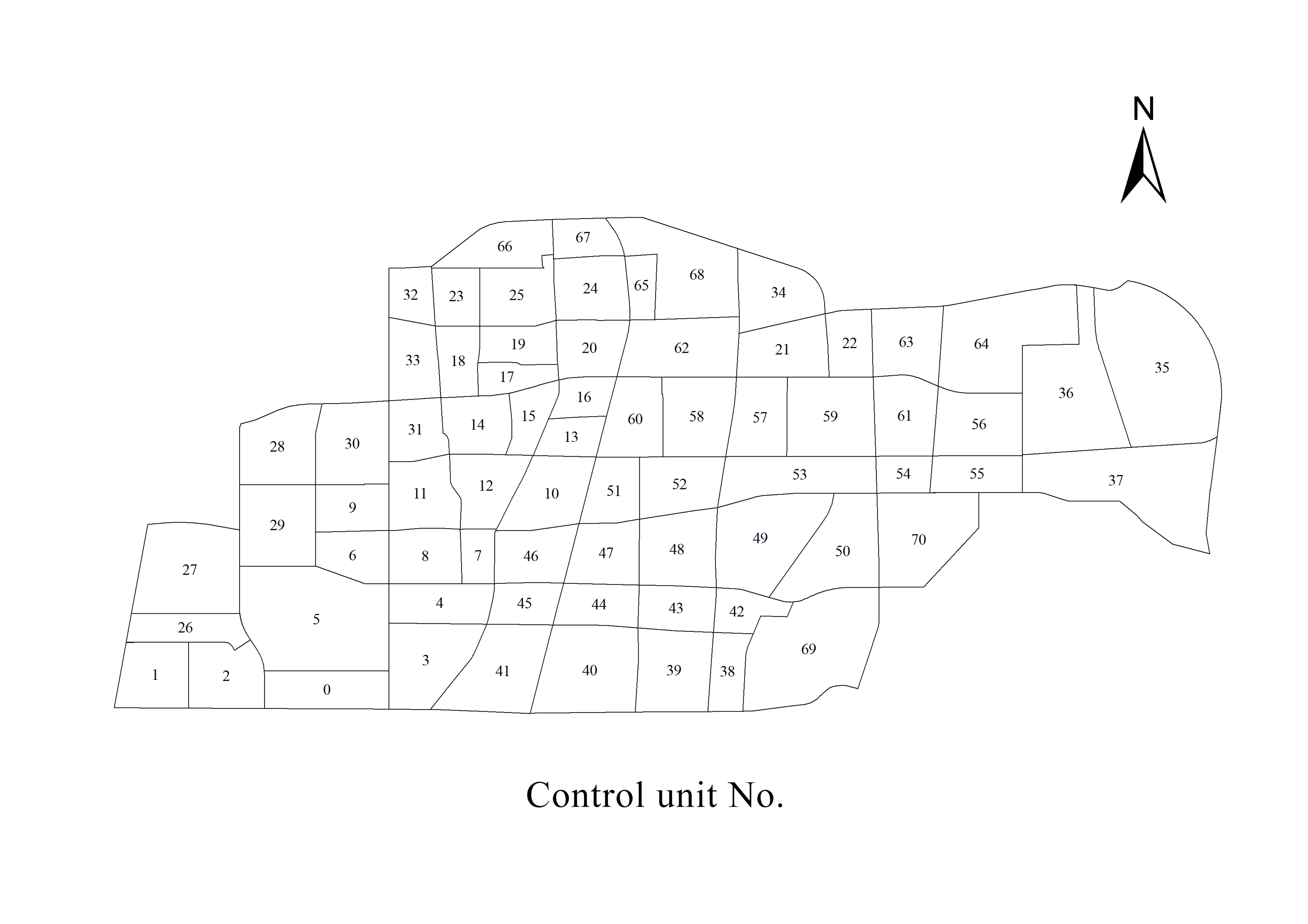

Supplement: S1 Fig — (TIF) [file pone.0279470.s001.tif]
