# Supplementary material for: Assessing the health impacts of the urban expansion of small cities in China: A case study of Jiawang
Source: PLoS One. 2022 Dec 22;17(12):e0279470. doi: 10.1371/journal.pone.0279470 (PMC9778500; doi:10.1371/journal.pone.0279470)
Supplement: S1 Appendix — (DOCX) [file pone.0279470.s003.docx]

**Appendix:** **Literature review for the framework of the health impact assessment**

| Factors | Health determinants | References | Health evidence | Influence path |
| --- | --- | --- | --- | --- |
| **Public facilities** | Proportion of green space | [1, 2] | [3-14] | obtaining public services, reducing negative health effects, promoting physical activity, promoting social communication |
|  | Per capita green space | [15, 16] | [3-14] | reducing negative health effects, promoting physical activity, promoting social communication |
|  | Service area of educational facilities (Kindergartens, primary schools and secondary schools) | [17, 18] | [19-21] | reducing negative health effects, promoting physical activity, |
|  | Accessibility to green space | [1, 22-25] | [3-14] | reducing negative health effects, promoting physical activity, promoting social communication |
|  | Accessibility to recreational facilities (Cultural facilities, sports facilities, squares and other types of facilities) | [1, 22-24] | [4, 26, 27] | obtaining public services, promoting physical activity, promoting social communication |
|  | Accessibility to healthcare facilities (Medical and health facilities, public welfare facilities, rehabilitation facilities) | [1, 22, 23] | [28] | obtaining public services |
|  | Accessibility to commercial facilities | [1, 22, 23] | [4, 29-31] | obtaining public services, promoting physical activity, promoting social communication |
| **Road transportation** | Urban road network density | [1, 18, 25] | [32] | reducing negative health effects, promoting physical activity |
|  | Intersection density | [2, 18, 24, 25] | (Norman et al., 2006) | reducing negative health effects, promoting physical activity |
| **Land use** | Land use mix | [1, 22, 23, 33, 34] | [35-38] | reducing negative health effects, promoting physical activity |
|  | Residential environment | [39] | [40, 41] | promoting physical activity |
|  | Residential density | [17, 22, 23, 25] | [42, 43] | promoting physical activity, promoting social communication |

**References:**

1. Ewing R, Schroeer W, Greene W. School location and student travel analysis of factors affecting mode choice. Transportation Research Record Journal of the Transportation Research Board. 2004;1895(1):55-63.

2. Handy SL, Boarnet MG, Ewing R, Killingsworth RE. How the built environment affects physical activity: views from urban planning. American Journal of Preventive Medicine. 2002;23(2-supp-S1):64-73.

3. Maas J, Verheij RA, Groenewegen PP, Vries SD, Spreeuwenberg P. Green space, urbanity, and health: how strong is the relation? J Epidemiol Community Health. 2006;60(7):587-92.

4. Vaz E, Cusimano M, Hernandez T. Land use perception of self-reported health: Exploratory analysis of anthropogenic land use phenotypes. Land Use Policy. 2015;46:232-40.

5. Ellaway A, Macintyre S, Bonnefoy X. Graffiti, greenery, and obesity in adults: secondary analysis of European cross sectional survey. BMJ (Clinical research ed.). 2005;331(7517):611-2.

6. Handy S, Cao X, Mokhtarian PL. Self-selection in the Relationship between the built environment and walking: empirical evidence from Northern California. Journal of the American Planning Association. 2006;72(1):55-74.

7. Tamosiunas A, Grazuleviciene R, Luksiene D, Dedele A, Reklaitiene R, Baceviciene M, et al. Accessibility and use of urban green spaces, and cardiovascular health: findings from a Kaunas cohort study. Environmental Health A Global Access Science Source. 2014;13(1):1-11.

8. Liu D, Kwan M, Kan Z. Analysis of urban green space accessibility and distribution inequity in the city of Chicago. Urban Forestry & Urban Greening. 2021;59(1):127029.

9. Wang L, Zhou Y, Wang F, Ding L, Love PED, Li S. The influence of the built environment on people's mental health: an empirical classification of causal factors. Sustainable Cities and Society. 2021;74:1-15.

10. Knobel P, Maneja R, Bartoll X, Alonso L, Bauwelinck M, Valentin A, et al. Quality of urban green spaces influences residents' use of these spaces, physical activity, and overweight/obesity. Environmental Pollution. 2020;271(224):116393.

11. Zhang W, Yang J, Ma L, Huang C. Factors affecting the use of urban green spaces for physical activities: views of young urban residents in Beijing. Urban Forestry & Urban Greening. 2015;4(14):851-7.

12. Yu H, Yang J, Li T, Jin Y, Sun D. Morphological and functional polycentric structure assessment of megacity: An integrated approach with spatial distribution and interaction. Sustainable Cities and Society. 2022;80.

13. Yu H, Yang J, Sun D, Li T, Liu Y. Spatial Responses of Ecosystem Service Value during the Development of Urban Agglomerations. Land. 2022;11(2):165.

14. Yu W, Yang J, Sun D, Yu H, Yao Y, Xiao X, et al. Spatial-Temporal Patterns of Network Structure of Human Settlements Competitiveness in Resource-Based Urban Agglomerations. Frontiers in Environmental Science. 2022.

15. Mobley LR, Root ED, Finkelstein EA, Khavjou O, Farris RP, Will JC. Environment, obesity, and cardiovascular disease risk in low-income women. American Journal of Preventive Medicine. 2006;30(4):327-32.

16. Powell LM, Chaloupka FJ, Slater SJ, Johnston LD, O'Malley PM. The availability of local-area commercial physical activity–related facilities and physical activity among adolescents. American Journal of Preventive Medicine. 2007;33(4 Suppl):S292-300.

17. Lee C, Moudon AV. Correlates of walking for transportation or recreation purposes. Journal of physical activity & health. 2006;3(s1):S77-98.

18. Nelson MC, Gordon-Larsen P, Song Y, Popkin BM. Built and social environments. American Journal of Preventive Medicine. 2006;31(2):109-17.

19. Broberg A, Sarjala S. School travel mode choice and the characteristics of the urban built environment: the case of Helsinki, Finland. Transport Policy. 2015;37(jan.):1-10.

20. Mcdonald NC. Children's mode dhoice for the school trip: the role of distance and school location in walking to school. Transportation. 2008;35(1):23-35.

21. Kerr J, Rosenberg D, Sallis JF, Saelens BE, Frank LD, Conway TL. Active commuting to school: associations with environment and parental concerns. Medicine & Science in Sports & Exercise. 2006;38(4):787-94.

22. Kockelman KM. Travel behavior as a function of accessibility, land use mixing, and land use balance: evidence from San Francisco Bay Area. Transportation Research Record Journal of the Transportation Research Board. 1997;1607:116-25.

23. Saelens BE, Sallis JF, Frank LD. Environmental correlates of walking and cycling: findings from the transportation, urban design, and planning literatures. Annals of Behavioral Medicine. 2003;25(2):80-91.

24. Li F, Fisher KJ, Brownson RC, Bosworth M. Multilevel modelling of built environment characteristics related to neighbourhood walking activity in older adults. Journal of Epidemiology & Community Health. 2005;7(59):558-64.

25. Coombes E, Jonesa AP, Hillsdon M. The relationship of physical activity and overweight to objectively measured green space accessibility and use. Social Science & Medicine. 2010;6(70):816-22.

26. Ellaway A, Lamb KE, Ferguson NS, Ogilvie D. Associations between access to recreational physical activity facilities and body mass index in Scottish adults. BMC Public Health. 2016;16(1):756.

27. Norman GJ, Nutter SK, Ryan S, Sallis JF, Calfas KJ, Patrick K. Community design and access to recreational facilities as correlates of adolescent physical activity and body-mass index. Journal of Physical Activity & Health. 2006;3(s1):S118-28.

28. Jones AP, Haynes R, Sauerzapf V, Crawford SM, Forman D. Geographical access to healthcare in Northern England and post-mortem diagnosis of cancer. Journal of Public Health. 2010;32(4):532-7.

29. Wang MC, Kim S, Gonzalez AA, MacLeod KE, Winkleby MA. Socioeconomic and food-related physical characteristics of the neighbourhood environment are associated with body mass index. J Epidemiol Community Health. 2007;61(6):491-8.

30. Handy SL, Clifton KJ. Local shopping as a strategy for reducing automobile travel. Transportation. 2001;28(4):317-46.

31. Li J, Lo K, Zhang P, Guo M. Relationship between built environment, socio-economic factors and carbon emissions from shopping trip in Shenyang city, China. Chinese Geographical Science. 2017;27(5):722-34.

32. Kelly-Schwartz AC, Stockard J, Doyle S, Schlossberg M. Is sprawl unhealthy? A multilevel analysis of the relationship of metropolitan sprawl to the health of individuals. Journal of Planning Education and Research. 2004;24(2):184-96.

33. Cervero R, Duncan M. Walking, bicycling, and urban landscapes: evidence from the San Francisco Bay Area. American Journal of Public Health. 2003;93(9):1478-83.

34. Ewing R, Schmid T, Killingsworth R, Zlot A, Raudenbush S. Relationship between urban sprawl and physical activity, obesity, and morbidity. American Journal of Health Promotion. 2003;18(1):47-57.

35. Oliver L, Schuurman N, Hall A, Hayes M. Assessing the influence of the built environment on physical activity for utility and recreation in suburban metro Vancouver. BMC Public Health. 2011;11(1):959.

36. Aytur SA, Rodriguez DA, Evenson KR, Catellier DJ, Rosamond WD. Promoting active community environments through land use and transportation planning. American Journal of Health Promotion. 2007;21(4s):397-407.

37. Frank LD, Pivo G. Impacts of mixed used and density on utilization of three modes of travel: single-occupant vehicle, transit, walking. Transportation Research Record Journal of the Transportation Research Board. 1994;1466:44-52.

38. Duncan MJ, Winkler E, Sugiyama T, Cerin E, DuToit L, Leslie E, et al. Relationships of land use mix with walking for transport: do land uses and geographical scale matter? Journal of Urban Health. 2010;87(5):782-95.

39. Balfour JL, Kaplan GA. Neighborhood environment and loss of physical function in older adults: evidence from the Alameda County study. American Journal of Epidemiology. 2002;155(6):507-15.

40. Babisch W, Ising H, Kruppa B, Wiens D. The incidence of myocardial infarction and its relation to road traffic noise— the Berlin case-control studies. Environment International. 1994;20(4):469-74.

41. Dunstan F, Fone DL, Glickman M, Palmer S. Objectively measured residential environment and self-reported health: a multilevel analysis of uk census data. PLoS ONE. 2013;8(7):e69045.

42. Xu F, Li J, Liang Y, Wang Z, Hong X, Ware RS, et al. Associations of residential density with adolescents' physical activity in a rapidly urbanizing area of mainland china. Bulletin of the New York Academy of Medicine. 2010;87(1).

43. Sarkar C, Webster C, Gallacher J. Association between adiposity outcomes and residential density: a full-data, cross-sectional analysis of 419 562 UK Biobank adult participants. The Lancet Planetary Health. 2017;1(7):e277-88.
